# Sudden cardiac arrest associated with myxedema coma due to undiagnosed hypothyroidism: a case report

**DOI:** 10.1186/s12902-021-00894-4

**Published:** 2021-11-17

**Authors:** Asami Yoshinaka, Masayuki Akatsuka, Shuji Yamamoto, Michiaki Yamakage

**Affiliations:** 1 Department of Anesthesiology, Obihiro Kosei Hospital, West 14, South 10, 080-0024 Obihiro, Hokkaido Japan; 2 Department of Anesthesiology, Sapporo Medical University School of Medicine, South 1West 16, Chuo-ku, 060-8543 Sapporo, Hokkaido Japan

**Keywords:** Myxedema coma, Cardiac arrest, Hypothyroidism, Case report

## Abstract

**Background:**

Myxedema coma, which occurs due to hypothyroidism, is a rare and life-threatening condition. Some patients have hemodynamic dysfunction, which consequently leads to cardiac arrest. The rarity of this condition makes it difficult to determine the cause of cardiac arrest. It is important to diagnose myxedema coma based on clinical findings, including physical examination and laboratory parameters. We present a case of undiagnosed and untreated hypothyroidism that initially caused myxedema coma and then led to cardiac arrest.

**Case presentation:**

A 56-year-old woman who had no medical history was transferred to our hospital for the management of return of spontaneous circulation due to sudden cardiac arrest. Findings of laboratory tests revealed that she had hypothyroidism. On physical examination, she was found to have a puffy face, thin eyebrows, and severe systemic non-pitting edema. Therefore, the patient was clinically diagnosed with myxedema coma, which was the cause of cardiac arrest. She was treated with thyroid hormone and hydrocortisone, resulting in improvement in her general condition, except for the neurological dysfunction.

**Conclusions:**

This case suggests that myxedema coma is caused by undiagnosed and untreated hypothyroidism, leading to sudden cardiac arrest. Our findings are useful in the differential diagnosis of hypothyroidism based on characteristic physical examination findings. Clinicians should be aware of the differential diagnosis of myxedema coma based on findings from physical examination and laboratory testing of thyroid function, and the treatment should be started immediately.

## Background

Hypothyroidism is a condition where serum thyroid hormone levels are lower than the reference range. If hypothyroidism is not identified early, it could lead to the most severe manifestation, known as myxedema coma. Myxedema coma is a rare form of extreme hypothyroidism, and it has been reported to occur at a frequency of 0.22/1,000,000 per year in Spain [1]. It is a life-threatening condition and is associated with high mortality rates [2–4]. In some cases, this condition could lead to cardiac arrest due to complications [5, 6]. When patients with sudden cardiac arrest (SCA) are managed by resuscitation and return of spontaneous circulation (ROSC), a differential diagnosis of hypothyroidism, including myxedema coma, should be considered.

Herein, we report a case of undiagnosed and untreated hypothyroidism resulting in myxedema coma and, consequently, cardiac arrest.

## Case presentation

This case study was conducted according to the CARE guidelines [7].

A 56-year-old woman had SCA. After ROSC, the patient was transferred and admitted to our hospital for the management of post-cardiac arrest syndrome in the intensive care unit (ICU). The patient had no particular medical history.

### Clinical findings

The patient had experienced general weakness and edema in the bilateral lower extremities for a few years, but the patient did not visit any hospital regarding this issue. Recently, the patient experienced poor appetite and ate almost nothing. Several days before the admission, the patient still had a poor appetite and could not carry out daily activities. The patient was unconscious at home and was brought to the hospital by the ambulance crew. Cardiac pulmonary resuscitation was performed by the ambulance crew, and ROSC was achieved. The patient was intubated and transferred to our hospital.

**Fig. 1:**
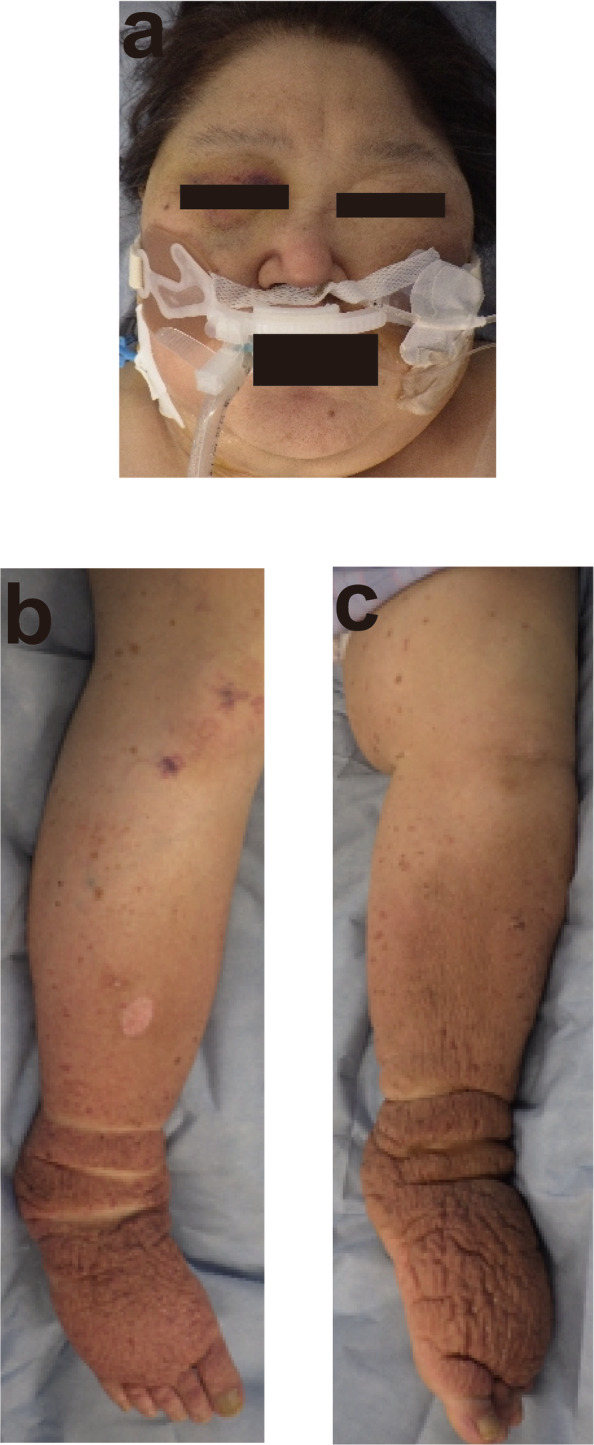
The patient’s appearance. **a** Puffy face, and thin eyebrows, **b**, **c** severe systemic non-pitting edema in the extremities

**Fig. 2:**
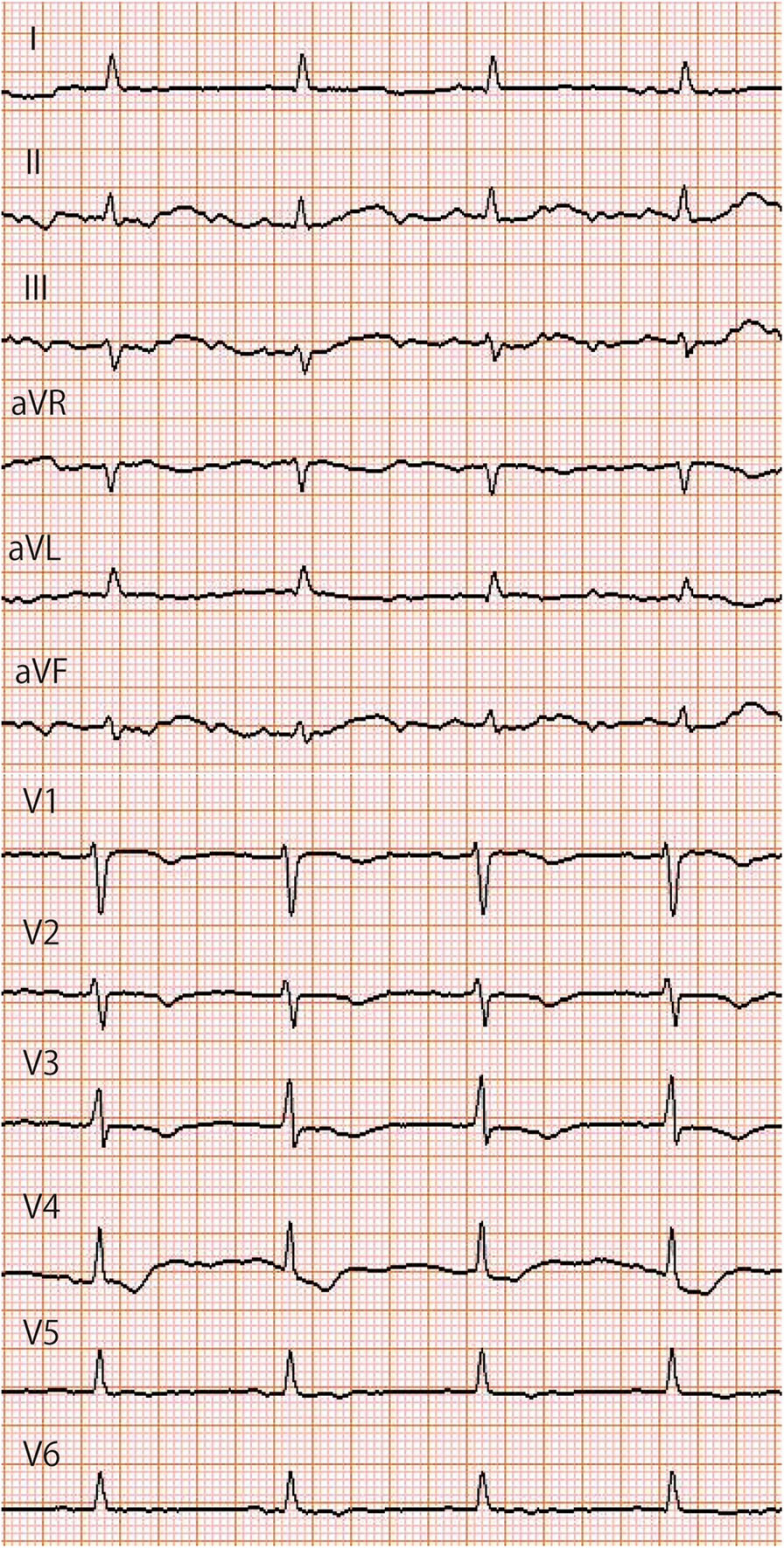
Electrocardiography. Electrocardiography shows low-voltage sinus bradycardia and an inverted T-wave in the anterior precordial leads

**Fig. 3:**
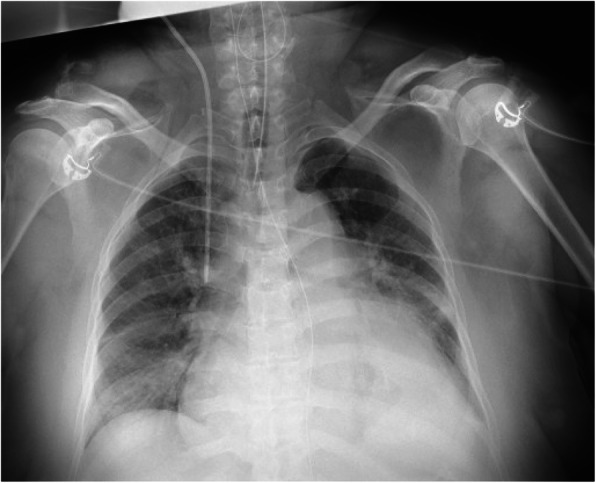
Chest radiography. Cardiomegaly and bilateral costophrenic angle blunting are noted

**Fig. 4:**
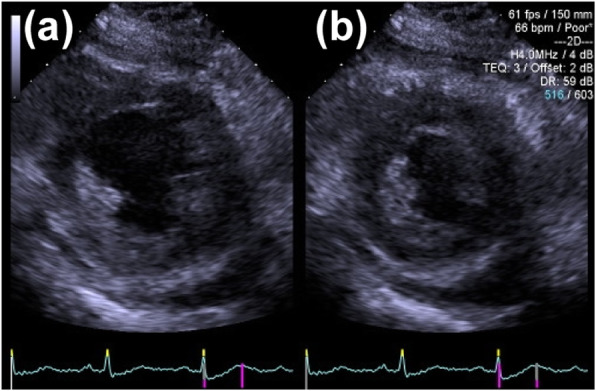
Echocardiography at short axis view. **a** Diastole **b** Systole. Hypokinesis of the inferior wall motion and a moderate amount of pericardial effusion are noted

**Fig. 5:**
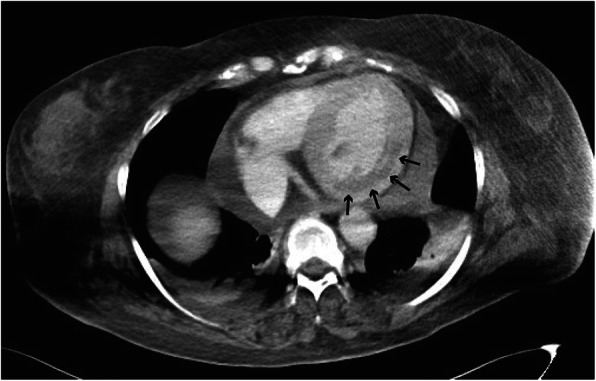
Contrast-enhanced computed tomography. In the contrast-enhanced computed tomography, there is no evidence of pulmonary embolism. However, a contrast-opaque area on the intimal side of the left ventricular inferior wall can be seen (indicated by the black arrows)

 Physical examination revealed a body mass index of 40.0 kg/m^2^. The initial vital signs on arrival at our hospital were as follows: Glasgow Coma Scale score of 6 (E1VTM4); blood pressure, 86/51 mmHg; heart rate, 55 beats/min; SpO_2_, 100 %; and respiratory rate, 21 breaths/min. The patient had hypothermia (core body temperature, 32.4 °C). On physical examination, the patient was found to have a puffy face, thin eyebrows, and severe systemic non-pitting edema (Fig. 1). Electrocardiography revealed low-voltage sinus bradycardia with an inverted T-wave in the anterior precordial leads (Fig. 2). Troponin I and brain natriuretic peptide levels were 1159 pg/mL and 138 pg/mL, respectively. Chest radiography showed cardiomegaly and bilateral costophrenic angle blunting (Fig. 3). Echocardiography, performed by a cardiologist, revealed hypokinesis of the inferior wall motion and a moderate amount of pericardial effusion (Fig. 4). Contrast-enhanced computed tomography showed no evidence of pulmonary embolism, but there was a contrast-opaque area on the intimal side of the left ventricular inferior wall (Fig. 5). Based on these findings, non-ST segment elevation myocardial infarction (NSTEMI) was suspected. The patient’s laboratory test findings were as follows: sodium, 125 mmol/L; potassium, 2.8 mmol/L; total protein, 6.1 g/dL; and albumin, 3.3 g/dL. Serum muscle enzyme levels were elevated, with a creatine kinase level of 1649 U/L and a lactate dehydrogenase level of 483 IU/L. Serum-free triiodothyronine (T3) level was 0.39 pg/mL, free thyroxine (T4) level was 0.04 pg/dL, and thyroid-stimulating hormone (TSH) level was 33.5 µIU/mL. The anti-thyroglobulin (Tg) antibody level was 3579 IU/mL, and the anti-thyroid peroxidase antibody level was 26.9 IU/mL. The serum cortisol level was 62.4 µg/dL, and the low-density lipoprotein-cholesterol level was 237 mg/dL.

### Treatment and outcomes

First, hydrocortisone was administered at a dose of 200 mg/day, considering the risk of adrenal insufficiency. Then, levothyroxine and liothyronine sodium were administered at doses of 200 µg/day and 50 µg/day, respectively. On the 5th day, the patient experienced sustained ventricular tachycardia (VT). Electrolyte levels were in the normal range, and the cause of VT could not be identified. The patient was clinically diagnosed with myxedema coma, which was possibly caused by NSTEMI. The patient received amiodarone at a dose of 750 mg for 48 h. Further cardiological intervention was not performed because the neurological prognosis was poor. After amiodarone administration, VT was not observed. On the 12th day, laboratory test results showed that the serum TSH level was 15.1 µIU/mL, the free T4 level was 0.21 pg/dL, and the free T3 levels were normal. However, the patient did not recover from the coma, and the dysfunction of the central nervous system remained.

Renal replacement therapy for acute kidney injury and antibiotic therapy for ventilator-associated pneumonia were needed during the stay in the ICU. On the 7th day, tracheostomy was performed, and management with mechanical ventilation continued. The patient was discharged to the chronic care hospital on the 25th day.

## Discussion and conclusions

This case study reveals the severe clinical course of myxedema coma.

Myxedema coma is a life-threatening and emergency presentation of hypothyroidism. However, because of its rarity, only a few cases of cardiac arrest on hospital arrival have been reported [5, 6]. Most previous studies have focused on the general clinical course of myxedema coma. However, in this case study, we report the characteristic physical examination findings for enabling the early diagnosis of myxedema coma caused by hypothyroidism.

A previous analysis [8] was conducted to reveal the epidemiology of myxedema coma in Japan. According to the study, the mean age at onset was 77 years, and two-thirds of the patients were women. The overall in-hospital mortality rate was 29.5 %. The number of patients was highest in winter. In-hospital mortality was significantly associated with higher age and the use of catecholamines. Cardiovascular diseases were the most frequent comorbidity at admission, which may be caused by hypothyroidism, leading to dyslipidemia, atherosclerosis, myocardial fibrosis, and cardiovascular injury. Several studies have used a scoring system for diagnosing myxedema coma [9, 10], but there are no global diagnostic criteria for myxedema coma because of its rarity and sudden onset. The Japan Thyroid Association has announced the widely used original diagnostic criteria, which include hypothyroidism, central nervous system failure, hypothermia, hypoventilation, cardiovascular failure, and hyponatremia. Therefore, myxedema coma is often clinically diagnosed based on symptoms and clinical examination without waiting for laboratory test results [11]. In this case, we considered the possibility that the cause of the disturbance of consciousness was hypoxic-ischemic encephalopathy after cardiac arrest. This was one of the reasons why it was difficult to differentiate it from the central nervous system disorder included in the diagnostic criteria for myxedema coma. Therefore, we clinically diagnosed the patient with myxedema coma without waiting for a response to the hormone replacement therapy.

Several factors precipitating myxedema coma have been revealed, such as infection, myocardial infarction (MI), cold exposure, surgery [12], and the administration of sedative drugs [13]. Amiodarone also has a complex effect on the thyroid gland because its structure is similar to that of thyroid hormones [14, 15]. In this case, fT3 levels were slightly lower, and TSH levels were higher after amiodarone administration for VT; consequently, the dose of LT3/LT4 needed to be increased. As a consequence, physicians should be cautious of certain drugs leading to thyroid dysfunction when managing patients with a myxedema coma.

Owing to the rarity of this condition, the recommended strategy of thyroid hormone replacement therapy remains unclear; thus, it has merely been based on expert opinions and case reports. In Japan, patients with myxedema coma are commonly treated with LT4, with or without LT3 [8].

Thyroid hormones have many effects on the heart and the vascular system. The most common signs of hypothyroidism are bradycardia, mild hypertension, narrowed pulse pressure, and attenuated activity on precordial examination. Low cardiac output is caused by bradycardia, decreased ventricular filling, and decreased cardiac contractility. Systemic vascular resistance may increase by 50 %, and diastolic relaxation and filling are slowed. However, heart failure is rare because cardiac output is usually sufficient to meet the lower demand for peripheral oxygen delivery [16]. Hypothyroidism may result in accelerated atherosclerosis and coronary artery disease, presumably because of the associated hypercholesterolemia and hypertension.

In this case, the patient had prolonged undiagnosed severe hypothyroidism, which favored several risk factors for cardiac events. On the admission day, MI was expected to occur, precipitating myxedema coma in the patient. Consequently, hemodynamics may have deteriorated, leading to an episode of cardiopulmonary arrest (CPA). Fortunately, the patient’s general condition, including her hemodynamic status, improved remarkably with thyroid hormone replacement therapy regardless of the use of amiodarone for VT. Unfortunately, the patient’s neurological dysfunction did not recover.

In conclusion, this case presents the clinical course of CPA caused by undiagnosed myxedema coma; this condition is characterized by unique findings on physical examination. Our findings are useful for the differential diagnosis of hypothyroidism, especially a diagnosis based on characteristic physical examination findings, in the emergency department.

Clinicians should be aware of the differential diagnosis of myxedema coma based on findings from physical examination and laboratory testing of thyroid function, and the treatment should be started immediately to prevent the occurrence of complications.

## Data Availability

Data are available on request to the authors.

## Abbreviations

ED: Emergency department
ROSC: Return of spontaneous circulation
SCA: Sudden cardiac arrest
ICU: Intensive care unit
T3: Triiodothyronine
T4: Thyroxine
TSH: Thyroid-stimulating hormone
MI: Myocardial infarction
CPA: Cardiopulmonary arrest

## References

[1] Rodríguez I, Fluiters E, Pérez-Méndez LF, Luna R, Páramo C, García-Mayor RV (2004). Factors associated with mortality of patients with myxoedema coma: prospective study in 11 cases treated in a single institution. J Endocrinol.

[2] Klubo-Gwiezdzinska J, Wartofsky L (2012). Thyroid emergencies. Med Clin North Am.

[3] Dutta P, Bhansali A, Masoodi SR, Bhadada S, Sharma N, Rajput R (2008). Predictors of outcome in myxoedema coma: a study from a tertiary care centre. Crit Care.

[4] Yamamoto T, Fukuyama J, Fujiyoshi A (1999). Factors associated with mortality of myxedema coma: report of eight cases and literature survey. Thyroid.

[5] Salhan D, Sapkota D, Verma P, Kandel S, Abdulfattah O, Lixon A (2017). Sudden cardiac arrest as a rare presentation of myxedema coma: case report. J Commun Hosp Intern Med Perspect.

[6] Dixit NM, Truong KP, Rabadia SV, Li D, Srivastava PK, Mosaferi T (2020). Sudden cardiac arrest in a patient With myxedema coma and COVID-19. J Endocr Soc.

[7] Riley DS, Barber MS, Kienle GS (2017). CARE explanation and elaborations: reporting guidelines for case reports. J Clin EPI.

[8] Ono Y, Ono S, Yasunaga H, Matsui H, Fushimi K, Tanaka Y (2017). Clinical characteristics and outcomes of myxedema coma: analysis of a national inpatient database in Japan. J Epidemiol.

[9] Popoveniuc G, Chandra T, Sud A, Sharma M, Blackman MR, Burman KD (2014). A diagnostic scoring system for myxedema coma. Endocr Pract.

[10] Chiong YV, Bammerlin E, Mariash CN (2015). Development of an objective tool for the diagnosis of myxedema coma. Transl Res.

[11] Takamura A, Sangen R, Furumura Y, Usuda D, Kasamaki Y, Kanda T (2017). Diagnosis of myxedema coma complicated by renal failure: a case report. Clin Case Rep.

[12] Yafit D, Carmel-Neiderman NN, Levy N, Abergel A, Niv A, Yanko-Arzi R (2019). Postoperative myxedema coma in patients undergoing major surgery: case series. Auris Nasus Larynx.

[13] Dong BJ (2000). How medications affect thyroid function. West J Med.

[14] Hawatmeh A, Thawabi M, Abuarqoub A, Shamoon F (2018). Amiodarone induced myxedema coma: two case reports and literature review. Heart Lung.

[15] Elnaggar MN, Jbeili K, Nik-Hussin N, Kozhippally M, Pappachan JM (2018). Amiodarone-induced thyroid dysfunction: A clinical update. Clin Update.

[16] Klein I, Ojamaa K (2001). Thyroid hormone and the cardiovascular system. N Engl J Med.

